# An inventory of national surveillance systems assessing physical activity, sedentary behaviour and sport participation of adults in the European Union

**DOI:** 10.1186/s12889-021-11842-1

**Published:** 2021-10-07

**Authors:** Maroje Sorić, Kaja Meh, Paulo Rocha, Wanda Wendel-Vos, Ellen de Hollander, Gregor Jurak

**Affiliations:** 1 Faculty of Sport, University of Ljubljana, Gortanova 22, 1000 Ljubljana, Slovenia; 2 Faculty of Kinesiology, University of Zagreb, Zagreb, Croatia; 3 Portuguese Institute of Sport and Youth, Lisbon, Portugal; 4 National Institute for Public Health and the Environment (RIVM), Bilthoven, Netherlands

**Keywords:** 24-h movement behaviour, Physical activity questionnaire, Physical inactivity, Sitting, Sedentarism, Sedentary behaviour questionnaire, Healthy lifestyle, Physical activity measurement

## Abstract

**Background:**

Physical inactivity has been recognised as a global public health problem that requires concerted action. This calls for systematic physical activity (PA) surveillance as a mechanism for assessing the problem and evaluating the effectiveness of related policies. Because countries tend to design their policy measures based on national surveillance data, here we present an inventory of existing national surveillance systems on PA, sedentary behaviour (SB) and sport participation (SP) among adult population in all European Union (EU) Member States.

**Methods:**

As a part of the European Physical Activity and Sports Monitoring System (EUPASMOS) project, a questionnaire was constructed in the form of an on-line survey to collect detailed information on existing national surveillance systems on either PA, SB, or SP. National HEPA focal points from all 27 EU Member States were invited to answer the on-line questionnaire and data collection took part in the period May 2018–September 2019.

**Results:**

National monitoring of PA or SB or SP for adults has been established in 16/27 EU Member States, that host 33 different PA/SB/SP monitoring systems. Apart from 3 countries that are using accelerometers (Finland, Ireland and Portugal), surveillance is typically based on questionnaires. In most Member States these questionnaires have not been validated in the particular language and cultural setting. Next, specific domains and dimensions of PA, SB and SP assessed vary a lot across countries. Only 3 countries (the Netherlands, Portugal and Slovenia) are monitoring all three behaviours while covering most of the domains and dimensions of PA/SB/SP. Lastly, as half of the existing surveillance systems set an upper age limit, in 9/16 countries that are monitoring PA/SB/SP, no data for people older than 80 years are available.

**Conclusions:**

Systematic surveillance of PA is lacking among 11/27 EU countries, with even few monitoring SB and SP. Besides, existing surveillance systems typically fail to assess all dimensions and domains of PA/SB/SP with only three countries maintaining monitoring systems that encompass all three behaviours while covering most of the domains and dimensions of PA/SB/SP. Hence, additional efforts in advocacy of systematic PA surveillance in the EU are called for.

## Background

Owing to its strong associations with a range of chronic diseases and premature mortality, both low physical activity (PA) and high sedentary behaviour (SB) are widely recognised as a global public health problem that requires concerted action [1]. Furthermore, physical inactivity does not only affect individual health, but also burdens national economies with increasing health care costs and productivity losses. The most recent estimates reveal that direct health care costs of physical inactivity in Europe reach international $11.7 billion per year, in addition to $3.8 billion of productivity losses [2]. To address the physical inactivity pandemics, a series of strategic documents were adopted on both global and European level [3–9]. All these documents call for PA and physical fitness surveillance systems as a means for monitoring trends and evaluating the effectiveness of the action plans. Additionally, a need for a harmonised public health surveillance system of the European population has been identified to obtain comparable data across countries and align their policies and action plans [10]. With strong political and legislative support [11] focusing on adults, the European Union (EU) established the European Health Interview Survey (EHIS) coordinated by Eurostat and run by national statistical offices to meet this challenge [12]. Within EHIS also data on PA and SB are being collected with a sub-module through a short PA questionnaire (EHIS-PAQ). The EHIS-PAQ [13] consists of 8 items covering PA during work, transportation, and leisure time as well as muscle-strengthening exercise during a typical week. Still, it does not provide much detail on intensity of PA, and has been shown to have only moderate validity [14]. Moreover, EHIS survey relies on output harmonisation and does not mandate a consistent questionnaire across all Member States, resulting in non-equivalent and sometimes missing information for some aspects of PA or SB. Therefore, a comprehensive picture of population trends and regional variations of the levels of PA and sedentariness in the EU can be obtained only by combining information from cross-national European surveillance systems and national ones, especially in countries that monitor PA with more valid tools (e.g., accelerometers), as well as with information from countries that monitor sport participation (SP) as a very important domain of PA. This combination of sources would enable more precise identification of PA characteristics across regions and over time, as well as a better evaluation of policy actions.

Two large overviews on surveillance systems which include population-based measures of PA performed by the World Health Organisation (WHO) in 2015 [15] and 2018 [16] concluded that the vast majority of the EU Member States are monitoring PA. Yet, no effort was made to distinguish between international and national systems, nor was a systematic nature of data collection and usage required in the definition of surveillance. In addition, the Determinants of diet and physical activity study (DEDIPAC), which gathered information on international, European regional and national surveillance systems on diet, PA and SB in 11 countries, identified three multinational European surveys, and a number of national and regional surveys on PA and SB among adult population [17]. However, although this study offered a comprehensive overview of EU-wide surveillance systems, it provided data on national surveillance for only selected few EU Member States.

Hence, there is a need for a thorough inventory of national surveillance systems on PA and SB across the EU. Additionally, data on SP could enrich this overview since sport activities represent an important domain of PA and exhibit large positive effects not just on physical, but also on mental and social health. To bridge this gap, an inventory on existing national surveillance systems on PA, SB and SP for adults in all EU Member States was prepared within the European Physical Activity and Sports Monitoring System (EUPASMOS) project. This paper summarizes and discusses its main outcomes, contributing to the development of the framework for national surveillance systems in these areas.

## Methods

A web-based questionnaire was constructed to seek data on existing national surveillance systems on either: 1) PA; 2) SB; or 3) SP. The questionnaire was constructed specifically for the purpose of this study and consisted of 48 items. For each country data on number of different monitoring systems was collected as well as details of each monitoring system (e.g., years of monitoring, age span of participants, number of participants). In the next section, data on domains and dimensions of PA, SB and SP measured in each national monitoring system were collected through multiple-choice questions. The last section of the questionnaire inquired which of the PA questionnaires used had been formally validated and gathered information about the use of device-based assessment in surveillance of PA.

Prior to data collection, the questionnaire was pilot-tested among EUPASMOS members. Then, in May 2018, national focal points of the European network for the promotion of health-enhancing physical activity (HEPA Europe) from all 27 EU Member States were invited to answer the on-line questionnaire. HEPA Europe is a WHO/Europe network, established in 2005 with the mission to provide a forum for the advancement of HEPA research, policy and practice across the WHO European Region. In 2013, EU Member States have been requested by the European Commission to appoint national physical activity focal points to support the framework to monitor HEPA policies. After having resolved issues on missing or incomplete information, data gathering was closed in September 2019, with 20 Member States having filled out the online questionnaire. In total, 18 countries provided all the requested information, two countries offered partial information, and seven countries didn’t complete the online questionnaire at all. After that, we collected data for the remaining seven Member States and for two Member States which filled out the online questionnaire, but some data were incomplete (i.e., Austria, Belgium, Denmark, Finland, Germany, Greece, Italy, Slovakia and Spain) through the information available in the DEDIPAC study [17] and the WHO’s PA country factsheets [16]. Additionally, we consulted national experts from all mentioned Member States to get information equivalent to the inventory questionnaire.

All methods and procedures of this study were in accordance with the Declaration of Helsinki and were approved by the Ethics Committee of the Faculty of Sport at the University of Ljubljana (ID: 10/2018). Having fully informed the participants about the aims and procedures of the study, their verbal consent was obtained. As only administrative data were recorded, and no personal data were collected, written informed consent was not required.

### Eligibility criteria

First, for the purpose of this inventory a surveillance system was defined as *a systematic collection, analysis and interpretation of the health-related data* needed for the planning, implementation, and evaluation of public health practice. Only systems that regularly monitor population level of physical activity, sedentary behaviours or sport participation in adults (18+ years), regardless of the sector from which the system stems (e.g., health, education, industry etc.), were considered eligible. Single cross-sectional, cohort and intervention studies that contain data on PA/SB/SP were excluded. In addition, as countries tend to design their policy measures based on national surveillance data, only national monitoring systems, and not local, regional or pan-European ones (e.g., EHIS, Eurobarometer), were considered eligible for inclusion in this inventory.

## Results

**Fig. 1:**
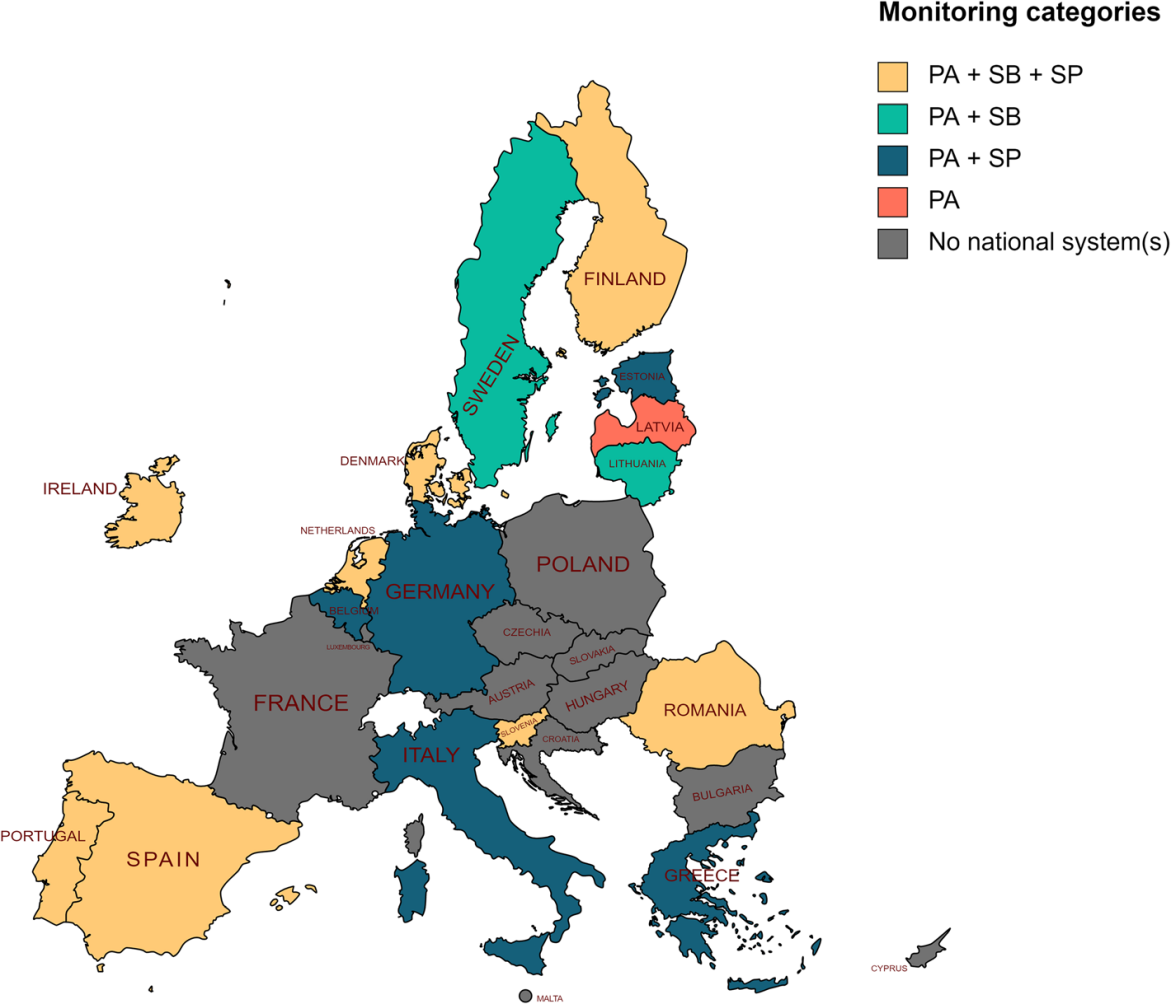
National PA/SB/SP monitoring systems across the EU

Figure 1 shows the availability of national PA/SB/SP monitoring systems across the EU. At the time of this study, national monitoring of PA, SB or SP for adults had been set up in 16/27 EU Member States (Belgium, Denmark, Estonia, Finland, Germany, Greece, Ireland, Italy, Latvia, Lithuania, the Netherlands, Portugal, Romania, Slovenia, Spain and Sweden). Sixteen Member States with established monitoring systems host 33 different PA/SB/SP surveillance systems. Nearly all these systems (*N* = 28) assess PA, while SP is evaluated in 15 national systems, and SB in 11.

Only 8 EU Member States are monitoring all three behaviours on a national level (Denmark, Finland, Ireland, the Netherlands, Portugal, Romania, Slovenia and Spain). In Belgium, Estonia, Germany, Greece and Italy, SB is not being monitored, while Lithuania and Sweden are not monitoring SP. Most national monitoring systems combine PA and SP (*N* = 10), or PA and SB (*N* = 8) in one survey. Twelve systems focus on only one of these behaviours (8 PA and 4 SP), while the Netherlands, Portugal and Romania assess all 3 behaviours in a single system.

A list of the monitoring systems and their general characteristics is provided in Table 1, while Table 2 summarises constructs, domains and dimensions covered, as well as the methods of assessment employed in each individual survey.

**Table 1:** Basic characteristics of movement behaviours monitoring systems across the European Union

***Country***, name of the system and (abbreviation)	Years of monitoring	Frequency of monitoring	App. number of participants	Age range of participants	Administration		Embedded or exclusively PA/SB/SP
					Mode	Means	
*Belgium*^a^							
Belgian National Food Consumption Survey (BNFCS)	2004, 2014	10 years	1200	18–64	Interview, Self-administered	Face to face interview, Computer-assisted	Embedded
BHIS	1997, 2001, 2008, 2013, 2018	5 years	10,000	19–64	Interview, Self-administered	Face to face interview, Computer aided personal interview	Embedded
*Denmark* ^a^							
Den Nationale Sundhedsprofil (DNS)	2010, 2013, 2017	3–4 years	520,000	16 +	Self-administered	Pen and paper, computer-assisted	Exclusively PA/SB/SP
Danskernes motions- og sportsvaner (DMS)	1964, 1975, 1987, 1993, 1998, 2002, 2004, 2007, 2011, 2016	Random	33,000	18 +	Interview, Self-administered	Face to face interview, Telephone interview, computer-assisted ^c^	Exclusively PA/SB/SP
*Estonia*							
Health Behaviour among Estonian Adult Population (HBEAP)	1993–2017	2 years	5500	18–74	Interview	Pen and paper	Embedded
National Physical Activity Survey among Estonian Adult Population (NPASEAP)	2015–2018	Annual	1000	15–69	Interview	Telephone aided personal interview	Exclusively PA/SB/SP
*Finland* ^a^							
National FinHealth Study (FinHealth)	2017 to date	5 years	7050	18+	Self-administered, Interview	Computer or online survey, Telephone interview	Embedded
Terveys 2011 (Health 2000–2011)	2000–2011	10 years	9000	18+	Self-administered, Interview	Pen and paper, Telephone interview	Embedded
Health Behaviour and Health among the Finnish Retirement-Age Population (EVTK)	1985–2013	Annual	2400	65–84	Self-administered	Pen and paper	Embedded
National FinSote study (FinSote)^d^	1987 to date	Annual	10,000	18+	Self-administered	Pen and paper, Computer or online survey	Embedded
*Germany* ^a^							
German Nutrition Survey (NVS II)	2005–2007	Random	14,291	14–80	Self-administered	Pen and paper	Embedded
German Health Interview and Examination Survey for Adults (DEGS)	2008–2011 (some participated in 98 GNHIES98)	Random	7988	18–79	Self-administered	Pen and paper	Embedded
*Greece* ^a^							
Sports for all programs (SFAP)^b^	1983, 1995 to date	Annual	60,000	5–80	Self-administered	Pen and paper	Embedded
*Ireland*							
Irish Sports Monitor (ISM)	2007, 2008, 2009, 2011, 2013, 2015, 2017	2 years	8500	16 +	Interview	Telephone interview	Exclusively PA/SB/SP
Healthy Ireland Survey (HIS)	2015–2018 to date	Annual	7500	15 +	Interview	Computer or online survey	Embedded
The Irish Longitudinal Study on Ageing (TILDA)	2009 to date	Annual	8000	50 +	Interview	Face to face interview	Embedded
*Italy* ^a^							
Aspetti della vita quotidiana (AVQ)	1993 to date	Annual	50,000/year	18–64	Self-administered	Pen and paper	Embedded
Progressi Delle Aziende Sanitarie per la Salute in Italia (PASSI)	2010 to date	Annual	35,000/year	18–69	Interview	Telephone interview	Embedded
Italian Population Survey on Alcohol and other Drugs (IPSAD)	2001–2009	2 years	85,000/year	15–64	Self-administered	Pen and paper	Embedded
*Lithuania*							
Study of the physical status of adults in Lithuania (SPS)	2010; 2014	5 years	5000	18–75	Interview	Pen and paper	Embedded
National Survey on Physical Activity in Lithuania (SPA)	2002, 2007, 2011, 2013–2017	Annual	1525	15–75	Interview	Computer aided personal interview	Exclusively PA/SB/SP
*The Netherlands*							
Health Survey/Lifestyle Monitor (HS/LSM)	1981 to date	Annual	10,000	4 +	Self-administered, Interview, Other	Computer aided web interview, computer-assisted telephone interview	Embedded
Additional module Physical activity and Accidents/Lifestyle Monitor (LSM-A PA & accidents)	2015 to date	2 years	10,000	4 +	Self-administered, interview, Other	Computer aided web interview, Computer-assisted telephone interview, Computer aided personal interview	Embedded
Dutch National Food Consumption Survey (DNFCS)	2003, 2005–2006, 2007–2010, 2010–2012, 2012–2016	2–3	depends on year	3 +	Self-administered	Pen and paper, Computer or online survey	Embedded
‘Omnibus of recreation’ (VTO)	2012 to date	2 years	3000	6 +	Self-administered, interview, Other	Computer or online survey, Computer aided personal interview	Embedded
*Portugal*							
National Physical Activity and Sport Monitoring System (NPASMS)	2006–2009 and 2017–2018	4 years	1250	18–64	Interview	Pen and paper	Embedded
*Romania*							
Study on behavioural determinants of health status for the adult population in Romania (CompSanRO)	2016 to date	2 years	1538	18–64	Interview	Telephone interview	Embedded
*Slovenia*							
Slovenian Public Opinion Survey (SPOS)	1973, 1975–76, 1980, 1983, 1986, 1989, 1992, 1996, 1997, 1998, 1999, 2000, 2001, 2004, 2006, 2008, 2012	Random	1250	15 +	Interview	Pen and paper	Embedded
Collaboration for Integrated Noncommunicable Disease Intervention (CINDI)	2001, 2004, 2008, 2012	Random	16,000	25–74	Self-administered	Pen and paper, Computer or online survey	Embedded
*Spain* ^a^							
Encuesta Nacional de Salud Española (Spanish National Health Survey) (ENSE)	1987–2017	Random	15,0260	15–85	Interview	Face to face interview	Exclusively PA/SB/SP
Consejo Superior de Deportes (CSD)	2005–2015	5 years	12,000	15 +	Interview	Telephone interview, Computer or online survey	Embedded
*Sweden*							
The national public health survey Health on equal terms (HLV)	2004–2016, 2018	2 years	40,000	16–84	Self-administered	Pen and paper, Computer or online survey	Embedded

Legend: ^a^ = countries with incomplete or missing data from the online questionnaire; ^b^ = the sample in the study is not random: ^c^ = depends on the year; ^d^ = FinSote study started in 1987 under the name Health Behaviour and Health among the Finnish Adult Population and was carried out till 2014 (conducted yearly for adults 18–64 years old, measured PA), after it became a part of The regional health and well-being survey carried out from 2010 to 2017 (conducted yearly for adults 20+ years, measured PA and SB), since 2018 FinSote study is carried out for 20+ adults, measuring PA; *PA* physical activity; *SP* sport participation; *SB* sedentary behaviour

**Table 2:** Domains and dimensions of movement behaviours covered by each monitoring system

Country, system	PAQ	Physical activity		Sedentary behaviour		Sport participation	
		Type & Domain	Dimension	Domain	Dimension	Domain	Dimension
*Belgium*							
Belgian National Food Consumption Survey	IPAQ-LF, EHIS-PAQ	Occupational, transport, cycling/walking, MVPA	Frequency, duration, intensity, total volume	–	–	Unspecified	Frequency, duration
BHIS	IPAQ-LF, EHIS-PAQ	Leisure-time, occupational, cycling/walking, MVPA,	Frequency, duration, intensity, total volume	–	–	–	–
*Denmark*							
Den Nationale Sundhedsprofil	NPAQ Marshall D	MVPA ^+^	Duration	Leisure-time, transport, school	Total volume	–	–
Danskernes motions- og sportsvaner	Newly developed/adapted	–	–	–	–	Organised, non-organised, competitive	Frequency, duration, type of sport
*Estonia*							
Health Behaviour among Estonian Adult Population	Newly developed/adapted	–	–	–	–	Unspecified	Frequency, duration
National Physical Activity Survey among Estonian Adult Population	Newly developed/adapted	Leisure-time	Frequency, duration	–	–	Unspecified	Frequency, duration, type of sport
*Finland*							
National FinHealth Study	Newly developed/adapted	Leisure-time, occupational, transport	Frequency, duration	–	–	Organised, non-organised	Frequency
Terveys 2011 (Health 2000–2011)	IPAQ-LF	Occupational, transport, home/household, cycling/walking	Frequency, duration, intensity, total volume	Leisure-time, occupational, transport	Screen time, sitting, total volume	–	–
Health Behaviour and Health among the Finnish Retirement-Age Population	Newly developed/adapted	cycling/walking, MVPA	Frequency, duration, intensity	–	–	–	–
National FinSote study^d^	Newly developed/adapted	Leisure-time, occupational, transport^c^	Frequency, duration, intensity, total volume^c^	Leisure-time, occupational^c^	Total volume^c^	–	–
*Germany*							
German Nutrition Survey	Newly developed/adapted	Transport, cycling/walking, home/household, gardening, house work, house maintenance	Duration	–	–	Unspecified	Other
German Health Interview and Examination Survey for Adults	Newly developed/adapted	MVPA	Frequency, duration	–	–	Unspecified	Duration
*Greece*							
Sports for all programs	Newly developed/adapted	Leisure-time	Frequency, duration	–	–	Organised	Frequency
*Ireland*							
Irish Sports Monitor	ISM	Leisure-time, transport, cycling/walking		–	–	Organised, non-organised, competitive, unspecified, coaching/volunteering	Frequency, duration, type of sport,
Healthy Ireland Survey	HIS	Leisure-time, transport, cycling/walking	Frequency, total volume	Total volume^c^	Sitting	–	–
The Irish Longitudinal Study on Ageing	IPAQ-SF	Leisure-time, transport, home/household, cycling/walking, coaching, volunteering	Frequency, duration, intensity, total volume	–	–	–	–
*Italy*							
Aspetti della vita quotidiana	Newly developed/adapted	PA	Frequency	–	–	Unspecified	Frequency, duration
Progressi Delle Aziende Sanitarie per la Salute in Italia	PASSI	PA at work and out of work	Frequency, duration, intensity	–	–	–	–
Italian Population Survey on Alcohol and other Drugs	IPSAD	PA	Frequency, duration, intensity	–	–	–	–
*Lithuania*							
Study of the physical status of adults in Lithuania	Adapted IPAQ-SF	Leisure-time, occupational, transport	Total volume	Leisure-time, occupational, transport	Total volume	–	–
National Survey on Physical Activity in Lithuania	GPAQ	Leisure-time, occupational, transport, home/household, cycling/walking	Frequency	–	–	–	–
*the Netherlands*							
Health Survey/Lifestyle Monitor	SQUASH	Leisure-time, occupational, transport, home/household, cycling/walking	Frequency, duration, intensity, total volume^c^	–	–	Organised, non-organised, competitive	Frequency, duration, type of sport, other
Additional module Physical activity and Accidents/Lifestyle Monitor	SQUASH Marshall D	Leisure-time, occupational, transport, home/household, cycling/walking	Frequency, duration, intensity, total volume^c^	Leisure-time, occupational, transport, school	Screen time, sitting, reclining/lying down, total volume	Organised, non-organised, competitive	Frequency, duration, type of sport, other
Dutch National Food Consumption Survey	SQUASH	Leisure-time, occupational, transport, home/household, cycling/walking, sport, gardening	Duration	–	–	–	–
‘Omnibus of recreation’	RSO	–	–	–	–	Organised, non-organised, competitivet	Frequency, type of sport
*Portugal*							
National Physical Activity and Sport Monitoring System	IPAQ-SF	Leisure-time, occupational, transport, home/household, cycling/walking	Frequency, duration, intensity, total volume	Leisure-time, occupational, transport	Screen time, sitting, reclining/lying down, total volume	Organised, non-organised	Frequency, duration, type of sport
*Romania*							
Study on behavioural determinants of health status for the adult population in Romania	National institute of public health questionnaire	Leisure-time, occupational, transport, cycling/walking	Frequency, duration, intensity	Leisure-time, occupational, transport	Total volume	Unspecified	Duration
*Slovenia*							
Slovenian Public Opinion Survey	The Sport-recreational activity of Slovenian	–	–	–	–	Organised, non-organised, competitive	Frequency, duration, type of sport
Collaboration for Integrated Noncommunicable Disease Intervention	Adapted IPAQ-SF	Leisure-time, occupational, transport, home/household, cycling/walking	Frequency, duration, intensity	Leisure-time, occupational, transport	Screen time, sitting	–	–
*Spain*							
Encuesta Nacional de Salud Española	IPAQ - SF	Leisure-time, occupational	Frequency	Leisure-time, occupational	Sitting	–	–
Consejo Superior de Deportes	IPAQ - SF	Leisure-time	Frequency, duration			Organised, non-organised, competitive	Frequency, duration, type of sport
*Sweden*							
The national public health survey Health on equal terms	IPAQ - SF	MVPA	Duration, intensity	Total volume	Sitting	–	–

*Legend*.; ^c^ = depends on the year; ^d^ = FinSote study started as Health Behaviour and Health among the Finnish Adult Population and then The regional health and well-being survey; *MVPA* moderate and vigorous physical activity; *GPAQ* Global Physical Activity Questionnaire; *NPAQ* Nordic Physical Activity Questionnaire; *ISMSF* Indagine Statistica multiscopo sulle famiglie (Multi-purpose statistical survey on families); *Marshall D* Marshall Sitting Questionnaire; *SQUASH* Short Questionnaire to Assess Health enhancing physical activity; *RSO* Richtlijn voor Sportdeelname Onderzoek (Guideline for Sport participation Research)

National PA/SB/SP monitoring systems in the EU are equally often based on interviews and self-administered questionnaires. Two predominant modes of administration are pen and paper (*N* = 16) and computer or online surveys (*N* = 14), while other means are used less frequently: telephone interviews (*N* = 10), and face to face interviews (*N* = 8).

The age range of participants across national PA/SB/SP monitoring systems differs to a great extent. Generally, systems include participants starting from the age of 15–18, while two systems are measuring PA/SB/SP exclusively in elderly (Finland and Ireland). On the other side, most systems have set the upper age limit for participation. Hence, in 9/16 countries that monitor PA/SB/SP no data for people older than 80 years are being collected.

Only seven countries are monitoring at least one of the behaviours annually (Estonia, Finland, Greece, Ireland, Italy, Lithuania, the Netherlands). In five countries PA, SB or SP are being monitored every 2–3 years (Denmark, Latvia, Romania, Slovakia and Sweden), while in Belgium, Portugal and Spain this is done less than every 3 years. Finally, Germany and Slovenia have reported irregular time intervals for monitoring PA/SB/SP.

Most of the national PA monitoring systems are embedded in larger surveys (*N* = 27), while Denmark, Estonia, Ireland, Lithuania and Spain run systems that focus exclusively on PA/SB/SP.

**Fig. 2:**
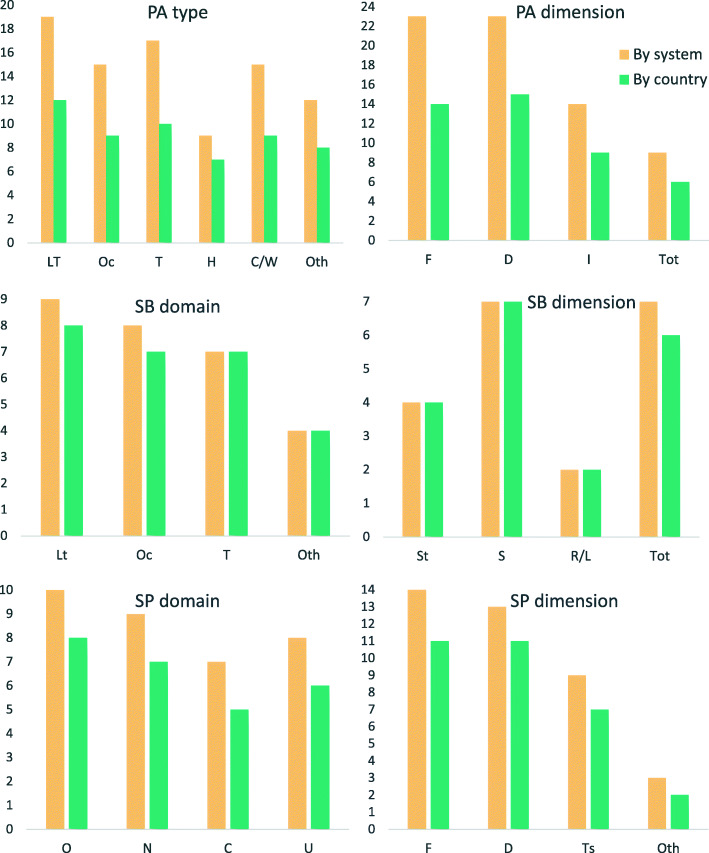
Specific domains and dimensions of PA/SB/SP included across monitoring systems and across countries. Note. Lt = leisure-time; Oc = occupational; T = transport; H = home/household; C/W = cycling or walking; Oth = Other; F = frequency; D = duration; I = intensity; Tot = Total volume; O = organised sport; N = non-organised sport; C = competitive/non-competitive sport; U = unspecified; Ts = type of sport; St = screen time; S = sitting; R/L = reclining/lying down

Figure 2 shows the number of systems and countries monitoring specific domains and dimensions of PA, SB and SP. It seems that PA and SB are most frequently monitored during leisure time, while the domains of SP are typically not specified. Frequency and duration are the most often reported dimensions of PA and SP, whereas sitting is the most commonly included dimension of SB. It should be noted that only the Netherlands, Portugal and Slovenia are running monitoring systems that encompass all three behaviours, while covering most of the domains and dimensions of PA/SB/SP.

Methods for assessing PA/SB/SP used in the identified monitoring systems are far from uniform. A lot of national PA systems are using custom questionnaires, developed specifically for their surveillance system (17 systems across 11 countries). Conversely, some countries rely on popular international questionnaires within their monitoring systems; most often on the International Physical Activity Questionnaire (IPAQ; used in 10 systems across 8 countries; mostly in short form). Most of the questionnaires being used needed to be cross-culturally adapted, but their measurement properties were not examined after the process. In effect, only six countries are using validated interpretations of original or adapted questionnaires in their national monitoring systems: Ireland, Portugal, Spain and Sweden had validated the IPAQ – short form, Denmark had validated the Marshall D questionnaire and the Netherlands had validated the SQUASH. Finally, only three countries (Finland, Ireland and Portugal) include device-measured PA (i.e., accelerometery) in their monitoring systems.

## Discussion

We gathered data about national systems that monitor PA/SB/SP of the adult population from all 27 countries in the EU. We identified different systems, but only analysed national, and not regional systems, and only included systems that were not a part of multinational surveys (e.g., EHIS). The main results of this study are: 1) PA, SB and SP of adults are still not being systematically monitored in 11/27 EU countries, while only 8 Member States are monitoring all three behaviours; 2) only the Netherlands, Portugal and Slovenia hold monitoring systems that encompass all three behaviours while covering most of the domains and dimensions of PA/SB/SP; 3) measurement tools being used show large heterogeneity across the countries with established monitoring systems; and 4) existing monitoring systems typically rely on PA questionnaires, while device-based assessment of PA/SB is scarce.

Although international surveillance systems enable between-countries comparisons, they typically lack detail and fail to consider cultural context of a specific environment. To this end, national systems that consider specific cultural contexts are needed for optimal policymaking. In a recent comprehensive analysis performed by the WHO, almost all EU Member States have been identified as having at least one surveillance system for PA [16]. In contrast, by excluding EU-wide surveillance (e.g., EHIS), and by insisting on more stringent inclusion criteria for surveillance (i.e., by including only systematic collection, analysis and interpretation of the health-related data and not sporadic, individual research studies) this study found that only a little over half of EU countries systematically monitor at least one of the concepts related to PA. Some of these monitoring systems date back to the twentieth century, as early as 1964 (Denmark) and 1973 (Slovenia), but most of the countries started monitoring PA in the 2000s. The growth of PA monitoring systems in EU coincides with an increase in PA promotion and other health-enhancing initiatives and policies [3, 4, 6–8]. Nevertheless, 11 Member States are forced to rely exclusively on estimates coming from EU-wide surveillance systems in the policy creation and policy evaluation process. These systems do not provide any information on domains or dimensions of PA, they typically lack detailed estimates across regions, and they are run only every 5–6 years. Although EU-wide systems are vital for policy making at the EU level, all these limitations impede their use for timely and efficient policy-making at a national level. In conclusion, much work is ahead if systematic and detailed surveillance of PA-related behaviours on a national level is to be achieved across the whole EU.

Next, we found that more than one half of existing surveillance systems exclude individuals older than 80 years. Considering that more than 27 million people older than 80 were living in the EU in 2016 [18], and that the share of elderly people has been projected to continue increasing, it is of paramount importance to also include the oldest age groups in the surveillance of PA/SB/SP. Besides the well-known benefits of PA for cardiovascular and metabolic health, in older adults PA has a vital role in the prevention of falls and falls-related injuries as well as in declines in bone and cognitive health.

Surveillance systems identified through this study typically included several domains of PA, most commonly leisure time and transport domains. At the same time, these two domains are the ones most amenable to interventions. With regard to the dimensions of PA, duration and frequency are being reported far more often than intensity. Although intensity of PA is the dimension that is the most difficult to assess, its importance has been repeatedly highlighted [19–21]. Unfortunately, even though the information about PA of different intensity is obviously available (e.g., from the IPAQ questionnaire), many surveillance systems fail to analyse and report these data. SB is monitored less frequently than PA, despite recent research showing the downsides of high SB and its deleterious effects on health [22–24]. Even when assessed, SB is typically conceptualised through only one dimension, mostly sitting, without any effort made to distinguish the context (e.g., watching TV, reading, playing, etc.). Given the accumulating evidence that unique sedentary behaviours relate to different health outcomes, including a number of SB dimensions should be encouraged. Next, in line with the recently introduced paradigm of 24-h movement guidelines [25], optimal surveillance should strive to include both PA and SB, while also assessing sleep. As global or EU-wide health surveillance systems (e.g., The Global Health Observatory, The European Health Interview Survey) do not include information on sleep duration, and since we did not collect data on sleep for national surveillance systems included here, it was not possible to identify countries in the EU that monitor all movement behaviours and are therefore able to assess compliance with the 24-h movement guidelines. Still, as we identified only 10 Member States that are monitoring both PA and SB, such estimates are evidently still not possible in the larger part of the EU.

Even though it has been well established that device-measured PA and SB are more reliable and valid compared to self-report [26–28], nearly all 16 EU countries with established national surveillance systems are relying exclusively on self-report for monitoring PA and SB, probably because they are economically and logistically more suitable for large surveys. Only three countries have reported monitoring PA with accelerometers (Finland, Ireland and Portugal). Yet, these assessments are not being performed on a regular basis and include a rather small number of participants. At the same time, several population-based research studies that had been set-up in the last few years and assessed PA via accelerometers in > 100,000 individuals, have shown that device-measured PA is feasible even on a large scale (e.g., UK Biobank study). Of note, the availability of device-based measures does not mean that the use of self-report methods should be discontinued. On the contrary, device-based assessment methods for PA/SB need to be accompanied with questionnaires that cover a wide range of domains and contexts of these behaviours in order to ensure the detail and the granularity of the data needed for data-informed policy making [29].

Surprisingly, many countries that have opted to perform cross-cultural adaptation of the existing questionnaires, have failed to confirm their measurement properties after the process. The fact that most countries are using questionnaires of unknown validity and reliability undermines the trustworthiness of conclusions based on the collected data and hampers the evaluation of the effectiveness of related policy measures.

Our study has several strengths. This is the first study to offer a very extensive overview of national surveillance systems for several movement-related behaviours across EU. First, we collected detailed data from all EU countries. Second, we extended our focus from PA to include other behaviours related to energy expenditure such as SB and SP. Finally, we analysed details such as domains and dimensions for all three behaviours.

Still, this study is not without limitations. First, despite several attempts we have not been able to contact seven countries via an online questionnaire (Belgium, Denmark, Finland, Germany, Greece, Slovakia and Spain), so the data had to be extracted from earlier studies or received through contact with independent national experts. As a result, some information for these 7 Member States might be incomplete or outdated. Second, we asked for details on the dimensions and domains of PA, but did not gather data on the type of PA. Hence, it was not possible to determine the extent to which national surveillance systems are able to evaluate muscle strengthening and balance elements of PA recommendations. Third, although we collected very comprehensive data on systems that monitor three distinct behaviours (i.e., PA/SB/SP), we did not request information on sleep surveillance. Consequently, we were not able to identify countries in the EU that are capable of evaluating adherence to the 24-h movement guidelines.

## Conclusion

Surveillance of PA is still not systematic in almost half EU Member States, and SB or SP are being monitored even less frequently. Besides, existing surveillance systems typically fail to assess all dimensions and domains of PA/SB/SP, and only three countries (the Netherlands, Portugal and Slovenia) run monitoring systems that encompass all three behaviours while covering most of the domains and dimensions of PA/SB/SP. Hence, additional efforts in advocacy of PA surveillance are needed. In order to facilitate the assessment of existing policies and optimise the effectiveness of new policies, countries should be encouraged to implement regular monitoring of PA with valid instruments for PA assessment. Moreover, embedding SB or 24-h movement behaviours assessment, together with SP, in existing and new surveillance systems should be advocated. The addition of device-based assessment methods for PA/SB to questionnaires that cover a wide range of domains and contexts of these behaviours would ensure the detail and the quality of the data needed for data-informed policy making. Finally, we are witnessing unprecedented restrictions in PA being implemented on a population level as a response to the current public-health crisis related to the COVID-19 pandemic. At the same time, work dynamics have transformed with the increase in remote work. As this is projected to reshape work environments in the long-term, monitoring occupational PA and SB will become more important. Comprehensive, continuous surveillance of all movement-related behaviours could have a vital role in creating and evaluating policies designed to combat the inactivity pandemics in the post COVID era.

## Data Availability

All data generated or analysed during this study are included in this published article.

## Abbreviations

DEDIPAC: Determinants of diet and physical activity
EHIS: European Health Interview Survey
EHIS-PAQ: European Health Interview Survey Physical activity questionnaire
EU: European Union
EUPASMOS: European Physical Activity and Sports Monitoring System
HEPA: Health-enhancing physical activity
IPAQ: International Physical Activity Questionnaire
PA: Physical activity
PAQ: Physical activity questionnaire
SB: Sedentary behavior
SP: Sport participation
SQUASH: Short Questionnaire to Assess Health enhancing physical activityWHO – World Health Organization

## References

[1] Bull FC, Al-Ansari SS, Biddle S, Borodulin K, Buman MP, Cardon G (2020). World Health Organization 2020 guidelines on physical activity and sedentary behaviour. Br J Sports Med.

[2] Ding D, Lawson KD, Kolbe-Alexander TL, Finkelstein EA, Katzmarzyk PT, van Mechelen W, Pratt M (2016). The economic burden of physical inactivity: a global analysis of major non-communicable diseases. Lancet..

[3] World Health Organization (2014). European food and nutrition action plan 2015–2020.

[4] World Health Organization (2016). Physical activity strategy for the WHO European region 2016–2025.

[5] Council. Council Recommendation of 26 November 2013 on promoting health-enhancing physical activity across sectors. Off J Eur Union. 2013;354(1):1–5.

[6] European Commission (2014). A growing health challenge for the EU. EU Action Plan on Childhood Obesity 2014-2020.

[7] World Health Organization (2018). Global action plan on physical activity 2018–2030: more active people for a healthier world [Internet]..

[8] World Health Organization (2013). Global action plan for the prevention and control of noncommunicable diseases 2013–2020.

[9] European Commission (2016). Report from the commission to the European parliament, the council, the European economic and social committee and the committee of the regions. Brussels.

[10] Finger JD, Tafforeau J, Gisle L, Oja L, Ziese T, Thelen J, Mensink GBM, Lange C (2015). Development of the European health interview survey-physical activity questionnaire (EHIS-PAQ) to monitor physical activity in the European Union. Arch Public Heal.

[11] European Parliament. Regulation (EC) no 1338/2008 of the European parliament and of the council of 16 December 2008 on community statistics on public health and health and safety at work. Off J Eur Union. 2008;12.

[12] Eurostat European Commission. European Health Interview Survey (EHIS wave 3) Methodological manual [Internet]. Publications Office of the European Union. Available online at: http://www.google.rs/url. Luxembourg: Publications Office of the European Union; 2018. 222 p. Available from: https://ec.europa.eu/eurostat/documents/3859598/8762193/KS-02-18-240-EN-N.pdf/5fa53ed4-4367-41c4-b3f5-260ced9ff2f6

[13] Baumeister SE, Ricci C, Kohler S, Fischer B, Töpfer C, Finger JD, Leitzmann MF (2016). Physical activity surveillance in the European Union: reliability and validity of the European health interview survey-physical activity questionnaire (EHIS-PAQ). Int J Behav Nutr Phys Act.

[14] Sember V, Meh K, Sorić M, Starc G, Rocha P, Jurak G (2020). Validity and reliability of international physical activity questionnaires for adults across EU countries: systematic review and Meta analysis. Int J Environ Res Public Health.

[15] World Health Organization. Factsheets on health-enhancing physical activity in the 28 EU Member States of the WHO European Region. Copenhagen: World Health Organization, Regional Office for Europe; 2015.

[16] WHO/Europe (2018). Physical activity country factsheets [Internet].

[17] Bel-Serrat S, Huybrechts I, Thumann BF, Hebestreit A, Abuja PM, De Henauw S (2017). Inventory of surveillance systems assessing dietary, physical activity and sedentary behaviours in Europe: a DEDIPAC study. Eur J Pub Health.

[18] Bourgeais V, Gereöffy A (2016). Nearly 27 million people aged 80 or over in the European Union. Eurostat Newsrelease [Internet].

[19] Laukkanen JA, Rauramaa R, Mäkikallio TH, Toriola AT, Kurl S (2011). Intensity of leisure-time physical activity and cancer mortality in men. Br J Sports Med.

[20] Amagasa S, Machida M, Fukushima N, Kikuchi H, Takamiya T, Odagiri Y, Inoue S (2018). Is objectively measured light-intensity physical activity associated with health outcomes after adjustment for moderate-to-vigorous physical activity in adults? A systematic review. Int J Behav Nutr Phys Act.

[21] Strain T, Wijndaele K, Dempsey PC, Sharp SJ, Pearce M, Jeon J, Lindsay T, Wareham N, Brage S (2020). Wearable-device-measured physical activity and future health risk. Nat Med.

[22] Ekelund U, Steene-Johannessen J, Brown WJ, Fagerland MW, Owen N, Powell KE, Bauman A, Lee IM (2016). Does physical activity attenuate, or even eliminate, the detrimental association of sitting time with mortality? A harmonised meta-analysis of data from more than 1 million men and women. Lancet..

[23] Madhav KC, Sherchand SP, Sherchan S (2017). Association between screen time and depression among US adults. Prev Med reports.

[24] Banks E, Jorm L, Rogers K, Clements M, Bauman A (2011). Screen-time, obesity, ageing and disability: findings from 91 266 participants in the 45 and up study. Public Health Nutr.

[25] Tremblay MS, Ross R (2020). How should we move for health? The case for the 24-hour movement paradigm. CMAJ..

[26] Skender S, Ose J, Chang-Claude J, Paskow M, Brühmann B, Siegel EM, Steindorf K, Ulrich CM (2016). Accelerometry and physical activity questionnaires-a systematic review. BMC Public Health.

[27] Prince SA, Adamo KB, Hamel ME, Hardt J, Gorber SC, Tremblay M (2008). A comparison of direct versus self-report measures for assessing physical activity in adults: a systematic review. Int J Behav Nutr Phys Act.

[28] Westerterp KR (2009). Assessment of physical activity: a critical appraisal. Eur J Appl Physiol.

[29] Sattler MC, Ainsworth BE, Andersen LB, Foster C, Hagströmer M, Jaunig J (2021). Physical activity self-reports: past or future?. Br J Sports Med.

